# Cognitive neuropsychological theory of antidepressant action: a modern-day approach to depression and its treatment

**DOI:** 10.1007/s00213-019-05448-0

**Published:** 2020-01-15

**Authors:** Beata R. Godlewska, Catherine J. Harmer

**Affiliations:** 1 Department of Psychiatry, Psychopharmacology Research Unit, University Department of Psychiatry (PPRU), University of Oxford, Oxford, UK; 2 Department of Psychiatry, Psychopharmacology and Emotion Research Laboratory (PERL), University of Oxford, Oxford, UK; 3 Oxford Health Foundation Trust, University Department of Psychiatry, Warneford Hospital, Oxford, OX3 7JX UK

**Keywords:** Cognitive neuropsychological model, Negative bias, Emotional processing, Antidepressant response

## Abstract

Depression is a leading cause of disability worldwide and improving its treatment is a core research priority for future programmes. A change in the view of psychological and biological processes, from seeing them as separate to complementing one another, has introduced new perspectives on pathological mechanisms of depression and treatment mode of action. This review presents a theoretical model that incorporated this novel approach, the cognitive neuropsychological hypothesis of antidepressant action. This model proposes that antidepressant treatments decrease the negative bias in the processing of emotionally salient information early in the course of antidepressant treatment, which leads to the clinically significant mood improvement later in treatment. The paper discusses the role of negative affective biases in the development of depression and response to antidepressant treatments. It also discusses whether the model can be applied to other antidepressant interventions and its potential translational value, including treatment choice, prediction of response and drug development.

## Introduction

Depression is a serious mental health condition, disabling and common, affecting around 20% of the population at least once in their lifetime (Ferrari et al. 2013).

Treatments for depression are available, and indeed help many sufferers (Cipriani et al. 2018). Unfortunately, finding the right medication is rarely a smooth process and multiple adjustments are usually needed; only about 30% of patients respond to their first treatment, and around 30% never recover and are considered treatment-resistant (Warden et al. 2007). These clinical observations stimulated intense research aimed at shortening the time to response. Two important themes emerged focusing on establishing treatment biomarkers to predict response and the development of new treatments that could help patients not responding to currently available ones.

Both psychological and biological aspects of depression have long been acknowledged, with clinical depression being defined as a result of complex interactions between psychological, behavioural and biological factors (Akiskal and McKinney Jr, 1973). However, individual studies have typically researched biological or psychological processes underlying depression and clinical response to treatments in relative isolation from each other. The past two decades however witnessed a change in research perspective. This was partly enabled by technological developments, allowing us to understand the biological underpinnings of the phenomena traditionally considered ‘psychological’, and vice versa, how biological interventions can affect ‘emotional’ entities. This has led to a better understanding of complex events occurring during the therapeutic process and opened the door for an exploration of novel treatment strategies, which would not be possible using a ‘biological’ or ‘psychological’ approach alone (Holmes et al. 2018).

Among technological developments, neuroimaging in particular expedited the collection of information on the mechanisms of depression and its treatments. The term ‘neuroimaging’ applies to a group of methods allowing a direct observation of the structure and the processes taking place in the living brain. One of its modalities, functional magnetic resonance imaging (fMRI), is particularly valuable in depression research as it allows a direct exploration of abnormal function—for example, response to salient visual and emotional information—as well as mapping of the abnormalities in particular structures and their networks of the brain. Its use has led to a fast increase in the understanding of core functional mechanisms of depression and effects of antidepressant treatments. Another functional modality—positron emission tomography—provides valuable information about brain metabolism and neurotransmitter function, though this has been less used due to its complexity and costs. Neuroimaging also allowed an exploration of brain structure and inter-structural connectivity through the use of, respectively, structural MRI and diffusion tensor imaging (DTI), and facilitated an insight into biochemical processes in the brain through magnetic resonance spectroscopy (MRS).

This review will consider new developments in the cognitive neuropsychological (CNP) hypothesis of antidepressant action, a model encompassing both biological and psychological elements, with a special focus on its potential translational applications. These developments were greatly supported by the use of fMRI as a tool.

## The cognitive neuropsychological hypothesis of antidepressant action

The cognitive neuropsychological hypothesis of antidepressant action (Harmer et al., 2009b, Godlewska, 2018) was developed in an attempt to understand a puzzling phenomenon of the delay in the appearance of the clear clinical effect of antidepressant drugs (ADs), which characterizes the majority of currently used antidepressant medications. This is counterintuitive as although the molecular, cellular and chemical effects occur very quickly after a drug gets administered, within the course of hours, symptomatic improvement is rarely seen before 1 week (Taylor 2007), with clear and sustained effects often not evident until 4–6 weeks of administration (Mitchell 2006; Frazer and Benmansour 2002). The CNP hypothesis has tried to elucidate the processes necessary for this symptomatic improvement.

**Fig. 1:**
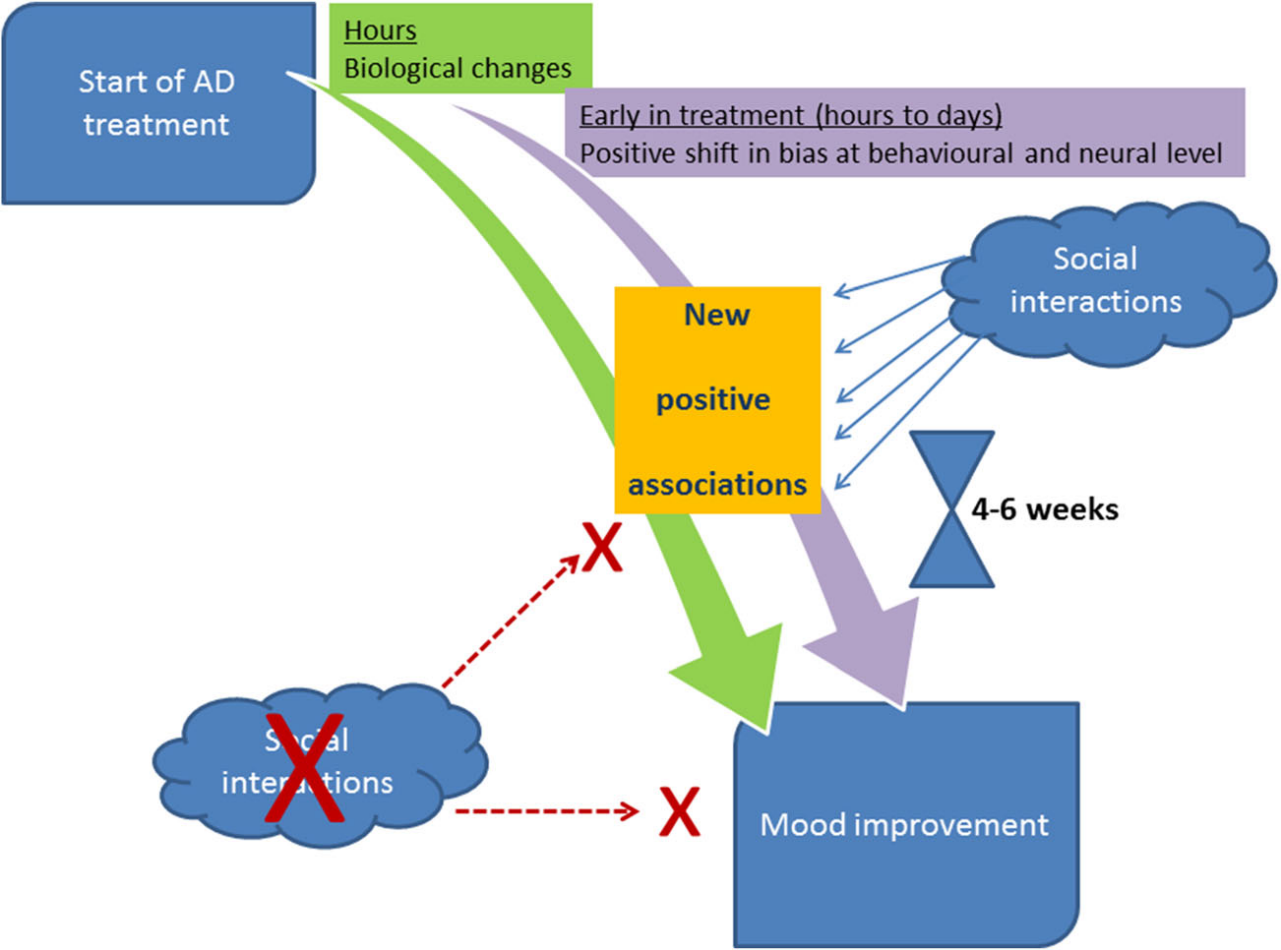
Schematic presentation of the cognitive neuropsychological model of antidepressant action. ‘Classical’ antidepressant drugs (ADs) (i.e. the ones that take weeks to induce mood change, such as selective serotonin reuptake inhibitors, SSRIs, or selective serotonin reuptake inhibitors, SNRIs, significantly improve mood only after a few weeks of treatment, despite biological changes taking place already in the first few hours after the first dose is applied, and continuing thereafter. The model proposes that for these early biological effects to be translated into clinical improvement, two key processes need to happen. The first one is a positive shift in the processing of emotionally salient information. Such a shift has been shown to occur at behavioural and neural level as early as after a single antidepressant dose. Subsequently, this new more positive bias needs to be ‘enacted’ by interactions with the social environment, which leads to the development of new positive associations in the learning associated processes. This happens in the context of continued downstream biological and neuroadaptive processes, for which repeated administration of ADs is needed. This is a lengthy process and explains the delay in the symptomatic improvement.

Embracing the idea of biological and psychological phenomena being strictly interlinked, the CNP hypothesis proposed a novel model in which the translation of initial biological effects of ADs into clinical response is mediated through their impact on negative bias in the processing of emotionally salient information. The hypothesis proposes that remediation of negative bias is crucial for clinical response, and that it occurs very early in the course of treatment. However, for the clinical effect, this new more positive bias needs to be ‘enacted’ by interactions with the social environment, which leads to the development of new positive associations. This process takes time and experience and thereby explains the delay in symptomatic improvement. The CNP model is schematically presented in Fig. 1.

## The point of focus for the CNP model: negative bias in processing of emotionally salient information in depression

Negative bias in processing in depression was described already in the 1950s and since then has been considered one of its core features; it results in a vicious circle of negative feelings, thoughts and behaviour, which act as a trigger and a maintaining factor for depressive symptoms (Beck 1967; Beck 1976; Elliott et al. 2011). Changes in cognition in depression are present during processing of both emotionally salient information and mental activity that does not involve emotions, so-called hot and cold cognition (Roiser and Sahakian 2013). The CNP model focuses on aspects of ‘hot’ emotional processing involving affective bias; ‘hot’ cognition also encompasses reward processing, and hyposensitivity to reward and hypersensitivity to punishment have been a consistent finding in depression (Treadway and Zald 2011).

Negative bias is not unique to depression. Its role is well established in a range of anxiety disorders (Davey and Meeten 2016) as well as bipolar disorder (Miskowiak et al. 2018a) and schizophrenia (Marwick and Hall 2008, Potvin et al. 2016). Hence, changes in emotional processing may be of transdiagnostic relevance, highlighting a core process which can affect symptoms and function across disorders. Understanding how these processes predict response to treatments and across different symptom domains is a major challenge for future research efforts.

Negative bias in emotional processing seen in depression has been reliably demonstrated across domains of emotional cognitive processing, including perception, attention, memory, and reward (Roiser et al. 2012, Roiser and Sahakian 2013, Robinson and Roiser 2016). For example, people with depression are more likely to perceive and categorize ambiguous facial expressions as negative in the Facial Emotions Recognition Test (FERT); they show preferential memory for negative emotional information in emotional word-based memory encoding (ECAT) and immediate and delayed recall (EREC) tasks; take longer to name the colour of negative emotional words in the emotional Stroop task (i.e. pay more attention or fail to disengage), and are slower to respond to happy targets in the emotional Go/No-Go task (Roiser et al. 2012). These paradigms, reliably used to assess emotional processing, were also employed to test the validity of the CNP hypothesis, as described later.

**Fig. 2:**
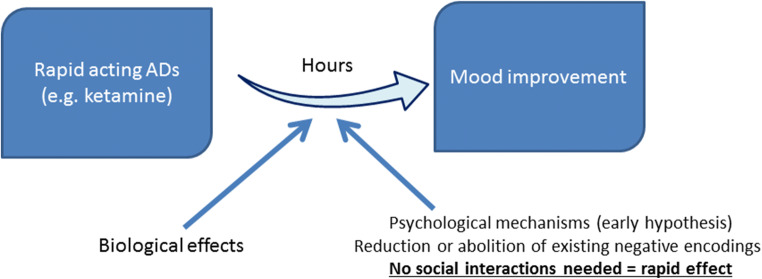
The hypothetical place of rapid acting ADs within the cognitive neuropsychological model of antidepressant action. Rapid-acting ADs, such as ketamine, were hypothesised to reduce or abolish memory for already existing negative associations. This, contrary to learning new positive ones, is a fast process, resulting in faster but more transient effect on mood. More research is needed to validate this hypothesis.

More recently, the development of modern technologies allowed an identification of the brain regions and networks relevant for affective processing. For example, during performance of the tasks designed to elicit responses to emotional stimuli—such as viewing facial expressions of fear, sadness or happiness—activity in the extended limbic system including the amygdala, pregenual anterior cingulate cortex (pgACC), and hippocampus, differed between depressed and healthy individuals, suggesting their role in the development and maintenance of emotional processing bias (e.g. Mayberg 2003) (Fig. 2).

Importantly, these findings fit with the proposed mechanisms of depression (Drevets 2001, Mayberg 2003). Research in humans, in particular using MRI as a tool, together with a large body of pre-clinical research in animal models using carefully designed assays based on objective measures rather than subjective experiences (Frazer and Morilak 2005), allowed the development of now widely accepted model of depression. In a schematic way, in a healthy state, reactivity of the structures responsible for fast automatic processing of emotionally salient information, such as the amygdala, ventral striatum and insula, is adequately controlled by the ‘higher’ structures, such as dorso-lateral prefrontal cortex (dlPFC), providing a balanced response appropriate to the weight of the emotionally loaded stimulus. The pgACC has an important place in these processes due to its central position within neural circuits involved in emotional and cognitive processing, which allows it to act as a crucial hub for the top-down regulation of initial limbic responses. In depression, this balance can be disturbed. The extended limbic system shows an increased response in many studies of depression, while the structures involved in regulation are hypoactive and therefore may be unable to exert the necessary control (Mayberg 1997, Drevets 2001, Mayberg 2003).

There is an agreement that both biological and psychological therapies remove the structural basis for negative bias formation, by affecting prefrontal and subcortical circuitry, however, possibly differing in terms of initial targets (Derubeis et al. 2008). Cognitive-behavioural therapy (CBT) is thought to directly target the cognitive processes and the negative schemata, this way strengthening the top-down control (Beck 1967, Shou et al. 2017, Yang et al., 2018). Medications, on the other hand, have been suggested to act on the structures directly involved in the negative bias formation, primarily on the extended limbic system (‘bottom-up’ effect) (Roiser et al. 2012). However, teasing apart these different effects has been challenging in human models as the response within top-down and limbic areas is correlated and effects of both medication and CBT have been observed at different loci within these networks.

For example, normalization of emotional processing at behavioural and neural levels was shown to take place during AD treatment and to correlate with clinical improvement (e. g. Sheline et al. 2001, Fu et al. 2007, Williams et al. 2015). Most studies explored changes before and after weeks of treatment, which undoubtedly increased the understanding of the mechanisms of AD action. At the same time, a question remained whether this positive shift was the cause or the consequence of mood improvement and which role it might play in treatment response. This question, with important practical implications, has become the key focus of the CNP hypothesis.

## Testing the validity of the cognitive neuropsychological hypothesis

Over the past decade, this theoretical model has been extensively researched.

Early research focused on healthy volunteers. Examining the effect of ADs in people without a history of depression—and other mental health conditions—was crucial for excluding the impact of typical symptoms of depression, such as low mood, memory and executive function deficits, on emotional processing and AD effect. Consistent with the model, antidepressants were applied for a short time, usually 1 or 7 days, which normally would not elicit any significant clinical response in depressed patients. After that study participants underwent behavioural testing and/or had an MRI brain scan while performing appropriate tasks, such as the aforementioned FERT, memory tasks or exposure to emotional facial expressions. The model was tested for medications representing different pharmacological groups, SSRIs, NRIs, atypical ADs, such as mirtazapine, as well as medicinal herb St John’s Wort. Later this research was extended to other antidepressant treatments, such as electro-convulsive therapy (ECT), discussed in more detail below.

In healthy individuals, short periods of treatment with the above medications were sufficient to produce a positive shift in emotional processing at behavioural and neural levels. Some differences between drugs were noticed. They seemed to reflect the primary mechanisms of action of these drugs. SSRIs tended to have a stronger impact on the processing of negative stimuli, in line with the putative role of serotonin (5-HT) in the development of negative affect. NRIs, on the other hand, had a stronger effect on processing of rewarding stimuli, which fits with the role for noradrenaline (NA) in the loss of positive affect and development of anhedonia (Pringle et al. 2011a).

**Fig. 3:**
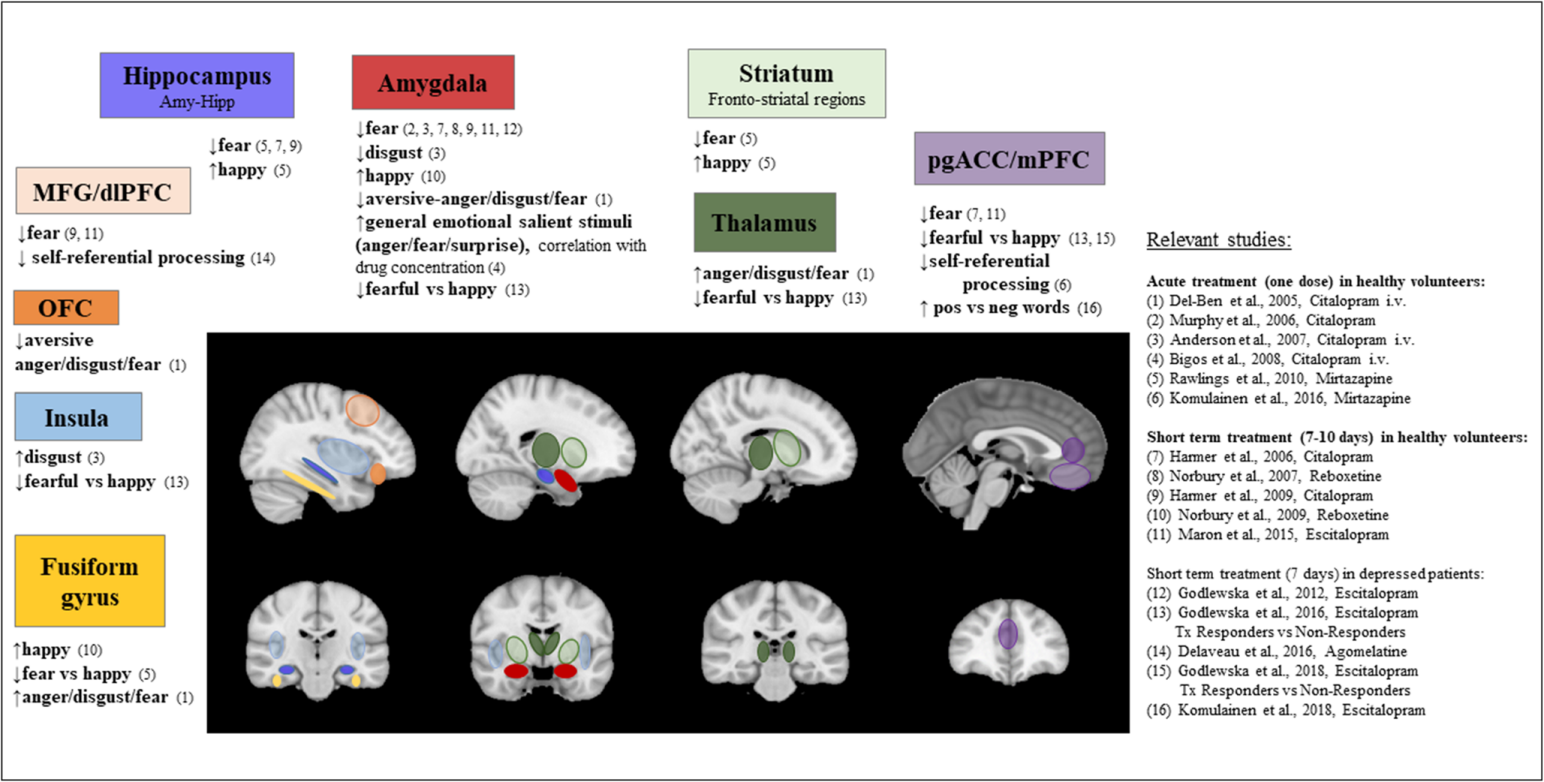
A schematic representation of changes in brain activity over an acute (one dose) or short-term (7–10 days) treatment with an antidepressant medication. Brain regions in which such changes were identified are listed in corresponding colour-coded boxes. Emotions, which processing in these regions has changed over treatment, and studies that showed these changes, are listed. Most of the studies used a variation of emotional faces processing task, the minority—self-referential processing task. Medications were applied orally unless otherwise indicated

ADs were shown to have an early effect on the neural networks dysfunctional in depression and proposed as the neural ‘scaffolding’ for the negative bias formation. Most consistent and robust reports regarded the amygdala and the ACC, but changes in response to emotionally salient information were also observed across other relevant structures, such as medial prefrontal cortex (mPFC), insula, ventral striatum, thalamus, orbito-frontal cortex and dlPFC (e.g. Harmer et al. 2004, Del-Ben et al. 2005, Harmer et al. 2006, Norbury et al. 2007, Anderson et al. 2007, Browning et al. 2007, Miskowiak et al. 2007, Bigos et al. 2008, Harmer et al. 2008, Harmer et al. 2009a, Murphy et al. 2009a, Arnone et al. 2009, Norbury et al. 2009, Rawlings et al. 2010, Anderson et al. 2011, Maron et al. 2016, Komulainen et al., 2016). A schematic representation of changes in brain activity over an acute (one dose) or short-term (7–10 days) treatment with various antidepressant medications is shown in Fig. 3. Figure 3 also shows the type of emotion affected by AD treatment and the direction of changes. Behavioural tests have shown broadly similar effects, with increased processing of positive vs negative stimuli across different measures of emotional processing with AD drug treatment (Harmer et al. 2003a, Harmer et al., 2003b, Murphy et al. 2006, Murphy et al. 2009b, Browning et al. 2007, Miskowiak et al. 2007, Harmer et al. 2008). Some variability was seen across the studies. The differences may be related to designs employed, such as the use of different drug doses, or way of drug application—oral or infusion—or additional factors such as baseline anxiety level. The short-term studies received strong support from a meta-analysis (Ma 2015), which explored fMRI response to AD treatments in healthy volunteers and patients with depression, administered as a single dose or repeated doses. This meta-analysis mapped changes into the brain regions identified in the short-term studies, including the amygdala, ACC, putamen, hippocampus, parahippocampal gyrus, thalamus, ventro-lateral PFC, dorso-medial PFC and dlPFC. The neuroimaging studies suggest that changes occur at the network level rather than in a single structure, although within these networks some areas may play a more important role and trigger more systemic changes (Dunlop et al. 2019).

Confirming the results of the meta-analysis in depressed patients and exploring whether the presence of the positive shift in emotional processing was necessary for clinical response was an important step in the validation of the CNP model. A recent fMRI study (Godlewska et al. 2016) showed a significantly larger effect of 7 days of treatment with an SSRI escitalopram on neural response to fearful versus happy facial expressions in people who after 6 weeks of treatment responded to the drug as compared to non-responders. This effect was seen across a number of structures being a part of neural ‘scaffolding’ for emotional processing, including the left amygdala, insula, anterior and posterior cingulate, bilateral supramarginal gyri and bilateral thalamus. Importantly, after 7 days of treatment, 6-week responders and non-responders did not differ significantly in terms of clinical improvement as measured by HAMD score. Although at day 7 reduction in the HAMD score was numerically higher in subsequent responders, its impact was excluded by secondary mediation analysis. This study reflected the results of an earlier behavioural study, which showed that an increase in the recognition of mildly intense happy facial expressions after 7 days of citalopram treatment predicted antidepressant response at 8 weeks. Interestingly, this was evident in people who perceived the level of their social support as adequate (Shiroma et al. 2014). These studies suggested that an early shift in emotional processing precedes mood improvement and is relevant for clinical response.

This conclusion was supported by results of a recent study on behavioural measures of emotional processing as a marker of future response (Browning et al. 2019). Seventy-four primary care patients with depression completed ECAT/EREC and FERT tasks, before treatment was started and after a week, with response to treatment assessed after 4–6 weeks. The best classifier from this group was then tested in another sample of 239 patients. The combination of a facial emotion recognition task and subjective symptoms was found to be the best predictor of response, with 77% accuracy in the training sample and 60% accuracy in the independent group.

These two studies are particularly important for the CNP hypothesis as they suggest an importance of an early positive shift in emotional processing for future response to treatment. At the same time, further work is needed to increase the accuracy with which this shift can predict the outcome.

Other studies have confirmed the presence of the positive shift in emotional processing early in the course of treatment, in the absence of significant changes in mood. Importantly, they also provided valuable information on two frequently raised concerns: the placebo effect and the impact of repeated testing. An fMRI study (Godlewska et al. 2012) showed that after 7 days of treatment with either 10 mg escitalopram daily or placebo, the amygdala response to fearful versus happy facial expressions was significantly higher in the placebo group as compared to the escitalopram group, while there was no difference between the escitalopram treated patients and healthy individuals. In the absence of mood differences between the placebo and escitalopram groups at baseline and after 7 days of treatment, this finding suggests that normalization of amygdala responsiveness was caused by medication, not placebo. Another study (Komulainen et al. 2018), comparing neural response after a week of SSRI escitalopram or placebo treatment, showed a decrease in response of medial fronto-parietal regions to self-referential emotional words relative to non-emotional control stimuli, and an increase in the mPFC and ACC to positive versus negative words. As in the previous study, there were no significant differences in mood between the two treatment groups, which again supports the effect of the drug, beyond the placebo effect. Another important concern is the impact of repeated testing. A study showing that neural response to fearful versus happy expressions differentiated between 6-week responders and non-responders to escitalopram (Godlewska et al. 2016), and that escitalopram treatment patients had a significantly larger neural response than the placebo group (Komulainen et al. 2018) suggested that even if the learning effect cannot be completely excluded, it does not account for the observed group differences. The results of these MRI studies are included in Fig. 3. A few other studies in depressed individuals added to this body of research. An increase in the recognition of happy facial expressions after 14 days of citalopram treatment (Tranter et al. 2009), as well as after a single dose and 14 days of reboxetine (Harmer et al., 2009a, Di Simplicio et al., 2014a) were shown. A shift in the recognition of other emotions—disgust and surprise—was seen after 14 days of treatment with both citalopram (Tranter et al. 2009) and reboxetine (Di Simplicio et al., 2014a). A recent study (Capitão et al. 2019) extended the findings into the population of adolescents with depression, showing that a single dose of fluoxetine, as compared to placebo, reduced reactivity in the amygdala-hippocampal region to angry facial expressions and increased activity in the dorsal anterior cingulate cortex (dACC) prior to changes in symptoms.

Observations in humans were supported by reverse translation of the findings into animal models, which opened new avenues for testing the effect of antidepressant drugs on core psychological processes. Effects of ADs were tested in rodents in induced negative affective states (Robinson and Roiser 2015) in the context of biases associated with reward learning and memory (Stuart et al. 2013) and biases of judgement in the presence of ambiguous information (Mendl et al. 2010, Hales et al. 2014). These studies have important implications for the CNP theory. For example, they facilitated a hypothesis regarding the mode of action of new fast-acting antidepressant drugs, discussed in more detail below (Stuart et al. 2015, Godlewska 2018).

Taken together, the above studies support the hypothesis of the role for an early positive shift in emotional processing in depressed individuals in future antidepressant response, and suggest that this shift precedes mood improvement. They also provide support for the effect of the medication beyond placebo and independence from the learning effect related to repeated testing.

## An essential part of the CNP hypothesis: interaction with the social environment

One of the assumptions of the CNP model is that although shift in emotional processing is crucial for clinical improvement, it is not sufficient for it to take place. Another key element is engagement in social interactions, which allows patients to relearn emotion-relevant associations in the more positive context created by the remediation of negative emotional bias. Although more research is necessary to test this prediction of the model, recent studies have provided it with some support. For example, in depressed individuals with late-life depression, an early bias shift was predictive of treatment response only in patients perceiving the level of social support as adequate (Shiroma et al. 2014). In particular, in a group of 27 depressed older adults, an improvement in recognition of mildly intense (25% and 50% intensity) happy expressions after 7 days of open-label treatment with an SSRI citalopram allowed prediction of remission after 56 days of treatment. Change in emotional processing was, however, a significant predictor of response and remission only when considered along with perceived level of social support, which was assessed using Interpersonal Support Evaluation List (ISEL), a list of 40 statements related to the perceived availability of potential social resources (Cohen and Hoberman, 1983). In line with this argument, training in negative bias seemed to affect mood only when people found themselves in stressful situations (MacLeod et al. 2002). Another study, in healthy volunteers, showed that treatment with citalopram, compared with placebo, protected against negative mood induction and that the extent of this protection was inversely correlated with the extent of shift in emotional memory bias (Browning et al. 2011). Further exploration of this important aspect of the CNP hypothesis is warranted, in particular in the light of its practical therapeutic implications, discussed below. Particular attention needs to be paid to designing the studies in a way that allows for testing interactions between changes in emotional processing and social or rewarding experiences.

## Can putative biological and psychological effects of ADs on emotional processing be integrated?

The notion that biological and psychological mechanisms of antidepressant effects on emotional processing are intrinsically linked is well supported by a growing body of evidence. Although the exact mechanisms, and in particular the precise timeline of events following antidepressant drugs application, are still to be elucidated, research already sheds some light on the processes that take place.

As most currently used ADs act—although often not uniquely—through the serotonergic system, a lot of attention has been directed towards this system. The role of 5HT in the modulation of the sensitivity and magnitude of neural responses in the brain regions involved in emotional processing is well established. Widespread projections exist from the dorsal raphe nuclei (DRN) to subcortical and cortical structures, including the amygdala, hippocampus, basal ganglia and OFC (Jacobs and Azmitia 1992, Hornung, 2003). It is not surprising that changes in 5HT levels induced by ADs have an effect on how emotionally salient stimuli are processed at the neural level.

The role of 5HT1A receptors has been explored in the context of the regulation of emotional processing. Lowered 5HT1A function in depression was shown in both pre-clinical and human studies. In humans, a direct exploration of the link between 5HT1A function and emotional processing was enabled by the use of PET. A negative correlation between DRN 5HT1A receptors availability and amygdala response to fearful facial expressions was shown (Fisher et al. 2006, Selvaraj et al. 2015), together with a positive correlation between DRN 5-HT1A availability and amygdala connectivity with the prefrontal-precuneus (ACC, middle frontal gyrus, supramarginal gyrus and precuneus) cortical network. These findings, even if a link is correlational, suggest a possibility that increased 5HT1A function is needed for appropriate control of emotional processing and that this control is exerted at the network level, including key structures for emotional processing. Interestingly, this link was shown only for fearful, and not happy, facial expressions, which could fit with the proposed role for 5HT in the generation and maintenance of negative emotional states.

Another effect of SSRIs is an increase in the production of neurotrophins, in particular, BDNF, which promotes neurogenesis and synaptogenesis in some of the key regions for emotional processing, in particular hippocampus (Gibon and Barker 2017). BDNF was shown to have an antidepressant effect in animal models of depression, for example, it increased time spent in the open arms of an elevated plus maze; mobility in the forced swim test; and sucrose consumption, and decreased latency in the novelty-induced hypophagia test (Phillips 2017). This neurotrophic effect could play an important role in consolidating early changes in emotional bias suggested by the CNP hypothesis and promote long-lasting influence of treatments.

## Are all interventions with antidepressant effect characterized by an early positive shift in emotional processing?

An early shift in emotional processing was shown across antidepressant medications classes. This naturally led to the question whether this phenomenon could be a feature of all AD treatments rather than a characteristic of a particular class of ADs or individual drugs, and whether it could be a mechanism through which they exert their clinical effect.

Cognitive Bias Modification (CBM) is an approach of particular interest in the context of the CNP theory as it directly targets putative psychological mechanisms of antidepressant action. CBM is designed to train attention away from the negative, and towards neutral or positive stimuli, without participant’s knowledge (CBM-A), or to modify the tendency to resolve ambiguity in a negative manner so that ambiguous information is interpreted as positive rather than negative. Despite initial promising reports regarding both CBM approaches (summarized in a narrative review by MacLeod et al., 2002), and claims of clinical benefits from both forms of CBM, a more recent meta-analysis (Cristea et al. 2015) failed to support this treatment effect, in particular in case of CBM-A. A possibility of some effect of CBM-I was suggested, however, not at the level that would match initial enthusiasm. Given the results of the above meta-analyses, it would be interesting to assess the efficacy of CBM in the context of social circumstances, which might play an important role in the response to this intervention, as suggested by the CNP hypothesis.

Possibly the best explored non-pharmacological antidepressant intervention in the context of the CNP model is electroconvulsive therapy (ECT). It was shown to induce a positive shift in emotional processing at the neural level already after a single session. This was manifested, for example, as a decrease in reactivity of the frontopolar cortex to retrieval of positive self-referential words (Miskowiak et al. 2018b), a decreased response in the mPFC to unpleasant vs pleasant pictures (Miskowiak et al. 2018c), or as a change in reactivity to fearful vs happy facial expressions in the parahippocampus and superior frontal gyrus (Miskowiak et al. 2017). All these studies emphasized no change in mood at the time of the assessment.

Novel therapeutic approaches that exert a neuromodulatory effect on the brain through the application of a magnetic or electric current via the scalp (Shiozawa et al. 2014) —respectively, transcranial magnetic stimulation (TMS) and transcranial direct current stimulation (tDCS)—have been shown to affect emotional processing. In healthy individuals TMS modulated neural activity in circuitry underlying emotion processing, and improved processing of emotional faces and emotional memory after a single session (De Raedt et al. 2010). A single session of tDCS was shown to modulate emotional processing in both healthy and depressed individuals, in the absence of mood changes (Nitsche et al., 2012, Brunoni et al. 2014, Ironside et al. 2016). In depressed volunteers, however, no relationship between bias changes with clinical response after 10 days was seen (Boggio et al. 2007). Deep brain stimulation (DBS) (Benabid et al. 2009), an invasive neuromodulatory approach, was shown to affect EEG components corresponding to positive shift in emotional bias 1 and 6 month after electrodes were implanted; EEG changes measured at month 6 negatively correlated with depression scores (Hilimire et al. 2015). Whether measurements at 1 and 6 months can be considered an early shift in emotional processing is debatable, but an effect of DBS on emotional processing is certainly worth attention.

Understanding neuromodulatory treatments in the context of the CNP model faces a conceptual challenge, due to their faster effect on mood than in case of conventional ADs. For example, about 50% of patients respond to ECT after three sessions and some as soon as after a single session (Fink 2014). Proposed theories include a larger magnitude of effect and hence a shorter period of interactions with the environment necessary for clinical improvement; or an early improvement in symptoms, such as a decrease in psychomotor retardation and an improvement in motivation during ECT treatment (Browning and Cowen, 1986), facilitating social interactions and more positive bias formation.

Another important group of treatments for depression are talking therapies. CBT, for example, is based on the premise that negative biases have a causal role in depression and makes them its therapeutic target. CBT has indeed been shown to cause a positive shift in emotional bias, both at the behavioural and neural level. It is hypothesised to work directly on the negative schemata and cognitive distortions and increase the top-down control in the executive and dorsal attention networks. Although this ‘top-down’ direction prevails, CBT has also been shown to have some ‘bottom-up’ effect, and decrease excessive reactivity of ventral cortico-limbic structures, similar to antidepressant medications (DeRubeis et al. 2008, Kalsi et al. 2017, Sankar et al., 2018). Due to complexity of CBT it is difficult to say which aspects of this treatment are the driving mechanisms of clinical change. An early shift in emotional processing during CBT treatment was shown in panic disorder (Reinecke et al. 2014) but has not been yet explored in depression.

## Translational value of the CNP model

Thanks to the latest technological developments, such as an introduction of various neuroimaging techniques, a better understanding of the interactions between phenomena traditionally considered ‘psychological’ and ‘biological’ became possible. It has become clear that they are not separate; that in fact they are closely linked and influence one another. The CNP model provided a theoretical frame for putting this new perspective in practice, allowing work on practical applications, as discussed below.

### Support for combination treatments

The CNP model provides support for combining biological and psychological/behavioural interventions.

As discussed above in more detail, the CNP hypothesis proposes that social interactions are crucial for newly formed positive bias to be translated into improvement in mood. This creates a framework to assess and predict the effects of combination treatments. Although ADs may induce a positive shift in emotional processing, in the lack of adequate social interactions this shift may not manifest itself further in the form of improved mood. Adding psychological and/or behavioural interventions to pharmacological treatments may help patients engage with their environment and experience stimuli necessary for creating and stabilizing new positive associations.

At the same time, a careful assessment is needed before making a decision which treatments should be combined. Some combinations, in theory working synergistically, may turn out to be less effective, or perhaps even harmful. For example, in a healthy volunteers study testing the effects of SSRI treatment and cognitive bias modification using computerized training alone and in combination, the combined treatment had a worse effect than each treatment in separation (Browning et al., 2011). Interestingly, applied on its own, cognitive bias modification training reduced the measures of depression recurrence (Browning et al. 2012).

### Biomarkers of clinical outcome and treatment personalization

The CNP model proposes an early shift in emotional processing as a putative surrogate marker of future clinical response. Surrogate markers need to be relatively easy to measure and reliably related to the outcome (Roiser et al. 2012). The importance of surrogate markers is particularly high when the outcome of treatment can be assessed only after a lengthy period of time. In such situations an early measurement of such biomarkers could markedly shorten the time to clinical response, by indicating a treatment best matched to an individual patient. For example, patients might start a treatment that usually, following clinical algorithms is attempted only after other treatments fail, as their first treatment.

The ease of assessment of emotional processing bias makes it a perfect measure to be used in the clinical setting. Behavioural test is particularly easy to apply, without the need for sophisticated equipment. At the same time, measurements at the neural level may be more reliable or sensitive. Given the burden of depressive symptoms, it would be certainly a cost-effective measure, especially with the growing accessibility and decreasing costs of scanning.

An early positive shift in emotional bias has been shown to differentiate between AD responders and non-responders and is a promising putative biomarker of clinical outcome, although not yet ready to be used in a wider clinical setting. Before this can happen, more work is needed to increase the accuracy of prediction, and to fit the model to differentiate between specific groups of drugs or individual medications. For now, an increased activity of pregenual ACC seems to be the most consistent general marker of good response to a variety of treatments, as supported by meta-analyses and shown in subsequent studies (Pizzagalli, 2011, Fu et al. 2013, Godlewska et al. 2016).

### Drug development

Another putative application of the CNP hypothesis is drug development. The model could help to decide which new agents may have antidepressant potential, and which should be abandoned.

#### A choice of agents to focus on

Given that an early positive shift in emotional processing was shown across a number of antidepressant treatments, a possibility that it may be used to detect agents with antidepressant potential is worth attention. This could be applied to new molecules in the early phase of their development or repurposing existing drugs. A use of this simple measure could allow cost-effective ‘fine tuning’ to the best treatment regime, by testing different doses or treatment periods in smaller groups of individuals, before running large clinical trials.

The model could potentially help to identify agents likely to act against particular symptoms, although this application needs more research to understand an impact of drugs on particular aspects of emotional processing, including processing of individual emotions. As briefly mentioned before, although a net effect of treatments with serotonergic and noradrenergic drugs is a positive shift in emotional processing, SSRIs seem to act primarily on the ‘negative’ aspects of emotional processing (e.g. decrease fear recognition and normalize exaggerated amygdala response to fearful faces), while NRIs are thought to primarily increase its ‘positive’ aspects (e.g. increase response to rewarding cues). This is in line with 5-HT dysfunction suggested to primarily result in the production of negative affect, i.e. sadness, and that of NA and DA, in a loss of positive affect and anhedonia (Pringle and Harmer 2015, Selvaraj et al. 2015). This knowledge might not only help with matching medications to individual patients’ symptoms, it might also help to define which symptoms the new compounds are likely to target, even when their mechanisms of action are not fully understood. Currently, the CNP model is being used to test medications that, based on their mechanism of action, have theoretical potential of being repurposed as antidepressants, such as 5HT4 antagonists or some anti-inflammatory drugs.

#### Elimination of agents without antidepressant potential from further research

Eliminating compounds that are not likely to show antidepressant effect despite preclinical data suggesting their role as ADs is crucial for directing attention and resources in the right direction. The CNP model could be a useful tool in this process, as suggested by research on memantine and aprepitant. Memantine, an NMDA antagonist used in the treatment of Alzheimer’s disease, failed to show AD effect in randomized controlled trials (Zarate Jr et al., 2006), despite promising results from animal research and an open-label trial in human participants. Interestingly, it also failed to affect emotional processing (facial emotion recognition and attentional vigilance to emotional words) and indeed potentiated the startle response (Sani et al. 2012, Pringle et al., 2012). Another putative antidepressant proposed on the basis of animal research was aprepitant, a neurokinin 1 (NK1) receptor antagonist. Its impact on emotional processing was inconsistent, with an effect on positive, but not negative aspects of emotional processing. It was shown to increase neural response in the ACC and amygdala to happy faces, but had no impact on fearful faces (McCabe et al. 2009); it increased vigilance towards all emotional words and recognition of happy facial expressions, but had no impact on fear recognition and categorisation or memory of emotionally valenced words (McCabe et al. 2009, Chandra et al. 2010, Pringle et al., 2011b). Interestingly, this echoed mixed support from earlier clinical trials (Keller et al. 2006; Kramer et al. 2004). Aprepitant never progressed into a licenced antidepressant medication.

#### Warning that a drug could have a negative impact

The CNP model might be particularly important as a tool warning against medications that are not only ineffective but actually harmful to patients. Such a medication was rimonabant, an inverse agonist of the cannabinoid receptor CB1, in the past used to treat obesity. It was removed from the market after repeated reports of the emergence of depressive symptoms, most worryingly suicidal ideations (Christensen et al. 2007). Interestingly, in healthy volunteers rimonabant, after a single dose and 7 days of treatment, was shown to have an effect opposite to what would be expected of an AD drug. It reduced positive bias, decreased the number of false recollections of positive words (Horder et al. 2012), and decreased the recall of positive words, with no effect on negative words (Horder et al. 2009).

## Way forward for the model: new avenues to explore

### Fast-acting glutamatergic drugs

The appearance of new fast-acting antidepressants, such as *N*-methyl-_D_-aspartate (NMDA) receptor antagonist ketamine, poses an interesting challenge for the CNP model. Ketamine acts on mood within hours and this time seems too short to allow an adequate level of social interactions, especially in the light of weeks needed for conventional ADs to work. One possible explanation could be that an impact on emotional processing by this group of drugs is different than in case of conventional ADs. This was supported by a translational rodent study (Stuart et al. 2015), which showed that ketamine might affect retrieval of already existing emotional memories i.e. abolish memory for negative associations rather than require creating new positive ones. This could result in faster but more transient mood improvement. In line with this, an fMRI study in depressed patients suggested that remediation of negative biases during ketamine infusion might be related to less interference of cognitive processing by negative emotional content (Scheidegger et al. 2016a). This hypothesis has been schematically presented in Fig. 2.

Interestingly, a 60 min infusion of two glutamatergic drugs, ketamine and lanicemine, a low trapping non-selective *N*-methyl-_D_-aspartate (NMDA) receptor antagonist, proposed as a ketamine-like antidepressant effect with fewer dissociative side-effects (Newport et al. 2015), increased reactivity of pgACC in those who showed symptomatic improvement 1 and 7 days later (Downey et al. 2016). This was in line with other studies showing that increased reactivity of pgACC was perhaps the most reliable general predictor of clinical response to pharmacotherapy and psychotherapy alike (Pizzagalli 2011, Fu et al., 2013, Godlewska et al. 2016). It was suggested that the first site of action for glutamatergic drugs might be ACC, and the treatment would shift it into a treatment-responsive mode in which it can restore balance between dorsal cognitive and ventral emotional processing pathways. Ketamine was also found to affect other elements of the networks involved in emotional processing. The findings included, for example, reduced neural reactivity in the bilateral amygdalo-hippocampal complex to emotional stimuli from the International Affective Picture System (IAPS), and correlation between reduced amygdala reactivity to negative pictures with resting-state connectivity to pgACC (Scheidegger et al. 2016b). These findings suggest that an early affective change may play a role in the mode of action of these drugs.

Further research on how glutamatergic drugs may affect emotional processing is needed before the CNP model can be reliably applied in this group of drugs.

### Exploring factors that may influence accuracy of prediction when using the CNP model

One of the important steps in improving sensitivity and specificity of the CNP model is to understand factors that may affect them. The need for such exploration was emphasized by a recent study showing that increased pretreatment dlPFC reactivity was predictive of future clinical improvement but only in people without a history of childhood abuse (Miller et al. 2015). If certain important factors are not taken into account, it may make the model less accurate in its predictive capacity. For the CNP model an obvious area to explore is interactions with the social environment; the accuracy may be affected if interactions are, for instance, not sufficient, negative or positive (e.g. Shiroma et al. 2014). There may be other factors which understanding in the future may refine the use of the model.

### ‘Fine-tuning’ of the CNP model

A change in emotional processing occurring over the first few days of antidepressant treatment may be a more complex process than initially expected and its relevance for treatment response is yet to be understood. For example, in healthy adults SSRIs, but not other drugs, were shown to increase fear recognition after a single dose, but not after 7 days of treatment. Interestingly, SSRIs often induce anxiety early in treatment. In so-called individuals with high neuroticism scores (healthy people with no clinical symptoms of depression, yet characterized by neural and cognitive bias in emotional processing typical for this condition), a single dose of SSRI citalopram increased recognition of positive emotions and shortened gaze maintenance at facial expressions (Jonassen et al. 2014), while 7 days of treatment increased amygdala response to both positive and negative emotions and elongated gaze maintenance (Di Simplicio et al. 2014b). This may reflect initial anxiogenic effect of SSRIs, abolished later on in treatment with improved engagement with social stimuli. In adolescents with depression a single dose of fluoxetine was shown to have a different early effect compared to adult populations, reducing neural response to angry faces, which fits with a decrease in irritation in this group of patients (Capitão et al. 2019). The timeline of the model might also need to be explored in more detail in the context of suggested onset of clinical effects way before the relevant effect is present, even after 1 week of treatment—for example, whether an early shift, as shown in some studies, puts some processes in motion. More research is needed to understand whether the sequence of changes in emotional processing may have an impact on their therapeutic effect.

### An impact of modification of individual emotions

Although there are no doubts regarding importance of positive shift in emotional processing in depression, it is currently impossible to state with certainty which emotion’s processing modification is crucial for therapeutic response. Based on a large body of research, in particular, behavioural studies, it might be tempting to see a shift in the processing of the negative effect as the key to successful AD effect, and a change in positive stimuli processing as less crucial; clinically this might be reflected by a greater efficacy of SSRIs over NRIs (Cipriani et al., 2018). At the same time, removal of negative affect in many cases may not be sufficient, which is illustrated by reports of emotional blunting as a result of SSRI administration or a failure to fully remediate the symptoms of anhedonia in depression (Goodwin et al. 2017). With SNRIs being a better choice in some patients, including those not responding to SSRIs, it seems plausible that a change in the processing of individual emotions may be less important than the ‘net’ effect of such changes. This was supported by one of our studies in depressed patients, in whom escitalopram modified processing of both ‘positive’ and ‘negative’ emotions, with a ‘net’ effect of a positive affective shift (Godlewska et al. 2016). This may, however, be a simplistic view of complex phenomena, in which things are further complicated by other factors, such as symptomatic make-up of a patient or the valence of environmental input in which people need to test newly acquired positive affective bias. This undoubtedly needs more research testing the effects of different treatments against each other in larger groups of patients.

## Conclusions

The CNP hypothesis of AD action has received substantial support from a number of well-designed and conducted studies. It embraces a modern approach to psychiatric conditions, viewing ‘biological’ and ‘psychological’ processes as interlinked, resulting from and influencing each other. It is a promising tool for a wide variety of clinically important applications, from treatment response prediction in individual patients to its use in the development of new antidepressant drugs. The ease of its use holds a promise that when it is ready, it could be easily adapted in both experimental and clinical settings.

## Abbreviations

i.v.: intravenous
pgACC: pregenual anterior cingulate cortex
mPFC: medial prefrontal cortex
MFG: middle frontal gyrus
OFC: orbito-frontal cortex
dlPFC: dorso-lateral prefrontal cortex.
